# Extracellular vesicles from ovarian cancer tumor spheroids harbor disease-related and survival-associated proteins

**DOI:** 10.20517/evcna.2025.70

**Published:** 2025-11-05

**Authors:** Christian Preußer, Max Gläser, Johannes Graumann, Witold Szymański, Daniel Bachurski, María Gómez-Serrano, Ralf Jacob, Silke Reinartz, Elke Pogge von Strandmann

**Affiliations:** ^1^Institute for Tumor Immunology, Philipps University Marburg, Marburg 35043, Germany.; ^2^EV-iTEC Core Facility, Philipps University Marburg, Marburg 35043, Germany.; ^3^Institute of Translational Proteomics and Core Facility Translational Proteomics, Philipps University Marburg, Marburg 35043, Germany.; ^4^Cluster of Excellence on Cellular Stress Responses in Aging-Associated Diseases (CECAD), University of Cologne, Cologne 50931, Germany.; ^5^Department of Cell Biology and Cell Pathology, Philipps University Marburg, Marburg 35043, Germany.

**Keywords:** Extracellular vesicles, ovarian cancer, HGSOC, primary 3D spheroids, tumor microenvironment

## Abstract

**Aim:** Extracellular vesicles (EVs) play a pivotal role in tumor progression, influencing the tumor microenvironment. Despite significant research, the targeted analysis of EVs directly derived from primary tumors remains limited, particularly in ovarian cancer. The majority of existing studies have focused on EVs derived from peritoneal fluid (ascites), which encompasses contributions from different cell types. This study aims to isolate and characterize EVs secreted specifically by ovarian cancer spheroids derived from primary patient ascites.

**Methods:** A three-dimensional cell culture model was employed to cultivate tumor spheroids in a defined medium, with EVs purified via differential ultracentrifugation and size-exclusion chromatography. Purified EVs were characterized by nanoparticle tracking analysis, nanoflow cytometry, and electron microscopy prior to performing high-resolution mass spectrometry.

**Results:** This approach allowed the identification of known cancer-associated proteins, including danger molecules, which are linked to poor prognosis. Moreover, enzyme-linked immunosorbent assay (ELISA) analysis demonstrated that the ascites abundance levels of novel candidates [RAB14 (Ras-related protein Rab-14), SCAMP3 (secretory carrier membrane protein 3), and FAM3C (FAM3 metabolism regulating signaling molecule C)] correlated with patients’ progression-free survival, further validating their clinical relevance. Finally, we used the Gene Expression Profiling Interactive Analysis 2 (GEPIA2) database to compare our dataset with The Cancer Genome Atlas (TCGA) and Genotype-Tissue Expression (GTEx) data. Thereby, we revealed a signature of three TOP genes encoding proteins within our dataset (CORO1B, LAMP2, MSLN), which were differentially expressed in ovarian cancer patients compared to healthy individuals.

**Conclusion:** This study provides the first proteomic profile of EVs derived directly from primary tumor spheroids, and paves the way for a better mechanistic understanding of EV-associated proteins and for the development of biomarkers or therapeutic strategies.

## INTRODUCTION

High-grade serous ovarian carcinoma (HGSOC) is the fifth most common cause of cancer-related mortality in women. While most tumors respond well to first-line chemotherapy, the 5-year survival rate is below 40% due to delayed diagnosis, early metastases, and the frequent development of therapy resistance^[1,2]^. In addition, the development of therapy resistance is a major factor contributing to disease progression and high mortality in HGSOC. This results in malignant ascites, representing a complex tumor microenvironment (TME) within the peritoneal cavity^[3,4]^. The TME is driven by interactions between tumor and host cells, creating a pro-tumorigenic and immunosuppressive environment^[5]^. Extracellular vesicles (EVs) are present in ascites and play a pivotal role in intercellular communication, modulating the TME. In malignant ascites, EVs form a heterogeneous population reflecting their diverse origins within the TME. These vesicles act as communication hubs, facilitating the distribution of tumor-derived factors throughout the peritoneal cavity^[6]^. Their heterogeneity underscores the challenge of identifying tumor-specific EV populations within the complex milieu of ascites. Plasma-derived EVs are more challenging to analyze, due to a lack of contribution of the localized TME and their commingling with vesicles from systemic, non-tumor sources. Despite the growing interest in EVs, research focusing on tumor-derived EVs in ovarian cancer is limited. Most studies to date have analyzed EVs from peritoneal fluid or plasma, which refer to mixed populations of vesicles from various cell types^[7]^. To date, few studies have examined EVs from primary tumor cells, which could provide more precise insights into their contribution to disease progression. To address this, we employed a novel approach by isolating primary tumor spheroids from patient-derived ascites and purifying their EVs for detailed analysis. Tumor spheroids represent a biologically relevant model of the primary tumor, recapitulating the hypoxic and nutrient-deprived conditions that drive aggressive cancer phenotypes^[8]^.

In this study, we present for the first time the characterization of primary tumor-derived spheroid EVs from patients with HGSOC. Our approach employs state-of-the-art methods to comprehensively analyze these EVs. We have gained a comprehensive understanding of the EV signatures of these vesicles by employing proteomic protocols. As a result, we screened for new markers in ascites not previously linked to ovarian cancer that may play a role in tumor progression and validated the association of the protein levels of RAB14 (a member of the RAS oncogene family), SCAMP3 (secretory carrier membrane protein 3), FAM3C (family with sequence similarity 3, member C) in ascites with progression-free survival (PFS). Using the Gene Expression Profiling Interactive Analysis 2 (GEPIA2) database, we established a signature of EV-associated factors that are differentially expressed between patients and healthy individuals and correlate with poor prognosis in ovarian cancer. This approach provides a more tumor-centric perspective and underscores the potential of spheroid-derived EVs as biomarkers and therapeutic targets. Moreover, it provides a foundation for advancing our understanding of EV-mediated communication in ovarian cancer and the relevance of EV-associated proteins in disease progression.

## METHODS

### Study approval

Ascites were collected from patients with HGSOC prior to their surgery at Marburg University Hospital. The collection and analysis of human materials were approved by the ethics committee of Philipps University Marburg (reference number AZ 205/10), and patients provided written consent before being included in the study.

### Spheroid isolation and culture conditions

Tumor spheroids were isolated from ascites as described earlier^[5,9]^. In brief, tumor spheroids were separated by filtration using a 30 μm filter. Spheroids and tumor single cells were purified by removing peritoneal leucocytes with CD45 microbeads and magnetic-activated cell sorting (MACS; Miltenyi Biotech, Bergisch Gladbach, Germany). The final tumor spheroids/cells had > 90% purity of EpCAM+ cells. Spheroids were cryopreserved at -80 °C. Spheroid cell pellets were directly resuspended in 5 µL pellet/ml in RPMI1640 (Gibco^TM^, Life Technologies, Carlsbad, CA, USA) without washing or centrifugation to maintain spheroid integrity and were cultured for 24 h in RPMI1640 medium with 10% FCS (Gibco^TM^) and 1% Penicillin/Streptomycin (Gibco^TM^) for recovery at 37 °C and 5% CO_2_. After 24 h, the medium was replaced with a CD293 medium (Gibco^TM^) - a chemically defined medium devoid of EVs, animal/plant/synthetic proteins, and undefined lysates or hydrolysates supplemented with GlutaMax (2:100) and 1% P/S (Gibco^TM^).

### Confocal microscopy

Spheroid cell pellets were resuspended in Matrigel (BD Biosciences; Heidelberg, Germany), added to precooled cover slips, and grown for the indicated time points. They were washed twice with phosphate-buffered saline (PBS) and fixed with 4% paraformaldehyde for 40 min. Afterwards, cells were permeabilized with 0.1% Triton-X-100 (Sigma Aldrich, Munich, Germany) for 20 min and blocked in 5% bovine serum albumin (BSA)/PBS for 2 h. Immunostaining was performed with anti-alpha tubulin in blocking reagents overnight. Secondary antibodies labeled with Alexa Fluor 488 (rabbit anti-goat Alexa Fluor^TM^ 488, Thermo Fisher, Waltham, MA, USA) were applied in PBS for 6 h. Nuclei were stained with Hoechst 33342. Following incubation, cells were washed with PBS and mounted with ProLongDiamond (Thermo Fisher; Waltham, MA, USA). Confocal images were acquired on a Leica STELLARIS equipped with a 93× glycerol planapochromat objective (Leica Microsystems; Wetzlar, Germany).

### EV isolation

EVs were isolated from spheroids by sequential centrifugation of the cell culture medium: 5 min at 300 × *g* (room temperature), 10 min at 2,000 × *g* (4 °C), and 45 min at 10,000 × *g* (4 °C), with the supernatant being transferred to a new tube after each step. The supernatants were then ultracentrifuged at 110,000 × *g* for 2 h at 4 °C in 14 mm × 89 mm Ultra-Clear^TM^ tubes using an SW41Ti rotor (Beckman Coulter, Krefeld, Germany; Optima XPN-80, k-factor 324). The resulting EV pellet was resuspended in 500 µL filtered 1× PBS (0.22 µm). For further purification, EV samples were subjected to size-exclusion chromatography: Sepharose CL-2B (GE Healthcare, Uppsala, Sweden) was packed into 10 mL polypropylene columns (Thermo Scientific, Rockford, USA) containing porous polyethylene discs (30 µm pore size), and equilibrated with filtered 1 × PBS. After loading 0.5 mL of EVs onto the column, fractions were eluted with filtered 1 × PBS (0.22 µm). EV-containing fractions (F8-10) were pooled and pelleted by ultracentrifugation for 2 h at 110,000 × *g* (4 °C) using a TLA45 rotor (Beckman Coulter, Optima Max-XP, k-factor 111).

### Nanoflow cytometry

For nanoflow cytometry (nFC) analysis, a Nano Analyzer (NanoFCM Co., Ltd., Nottingham, UK) equipped with a 488 nm laser was calibrated using 200 nm polystyrene beads (NanoFCM Co.) with a concentration of 2.08 × 10^8^ particles/mL, which also served as a reference for particle concentration. Monodisperse silica beads (NanoFCM Co. Ltd.) of four different sizes were used as reference standards to calibrate the size of EVs. Freshly filtered (0.22 µm) 1 × PBS was analyzed as a background signal and subtracted from all measurements. Each distribution histogram or dot plot was generated from data collected over 1 min at a sample pressure of 1.0 kPa. EV samples were diluted in filtered (0.1 µm) 1 × PBS to achieve a particle count within the optimal range of 2,500-12,000 events. Particle concentration and size distribution were determined using NanoFCM software (NF Profession v2.08, NanoFCM Co. Ltd.). For immunofluorescence staining, the following antibodies were used (BioLegend, Koblenz, Germany): fluorescein isothiocyanate (FITC)-conjugated mouse anti-human CD9 antibody (clone HI9a) and phycoerythrin (PE)-conjugated mouse anti-human CD63 antibody (clone H5C6). Isotype controls included FITC-conjugated mouse IgG1, κ (clone MOCP-21) at a concentration of 2 ng/µL in 100 µL 1 × PBS. After being centrifuged at 12,000 × *g* for 10 min to remove antibody aggregates, the supernatant was added to 2 × 10^8^ purified EVs. The mixture was incubated overnight at 4 °C with continuous shaking, then washed with 1 mL of filtered 1 × PBS (0.22 µm) by ultracentrifugation at 110,000 × *g* for 60 min at 4 °C (Beckman Coulter, Optima Max-XP, k-factor 111). The pellet was resuspended in 50 µL of 1 × PBS for nFC analysis.

### Electron microscopy

Formvar-coated copper grids (Science Services, Munich) were loaded with 5 µL of undiluted EV sample and incubated for 20 min. Grids were blocked for 30 min using Aurion blocking solution (#905.001), and then washed three times for 2 min in PBS containing 0.1% BSA-c (pH 7.4; Aurion, #900.099). Grids were fixed in 1% glutaraldehyde (Sigma, #G5882-100ML) in PBS for 5 min, followed by three washes in ddH_2_O for 2 min each. Negative staining was performed with 1.5% uranyl acetate (Agar Scientific, #R1260A) for 4 min before blotting. For each sample, an unstained control grid was prepared. Images were acquired using a Gatan OneView 4K camera on a JEM-2100Plus (Jeol) at 200 kV and analyzed with ImageJ (NIH).

### Mass spectrometry

EVs were lysed in 4.5 M urea, 1.5 M thiourea in 10 mM HEPES, pH 8.0, and subjected to an in-solution digestion and tandem mass tag labeling as described^[10]^, with the following modifications: Lys-C and trypsin were used at fixed amounts of 1.5 and 3 μg, respectively, and peptides were desalted using STAGE tips prior to labeling. Given divergent and minute peptide yields from the samples, isobaric labeling was performed using 5 samples each, along with a carrier channel from a particularly abundant sample (4 plexes). This approach served to boost the signal-to-noise ratio for peptide identification, and reporter ions were used solely to confirm peptide presence in a sample/channel and carry no quantitative information. Parametrization for the subsequent liquid chromatography/tandem mass spectrometry was extracted and summarized using MARMoSET (Metadata Automated Reporting for Mass Spectrometry Experiments and Tools)^[11]^. MARMoSET is an open-source utility that automatically compiles instrument and acquisition parameters from raw data files and is provided in the Supplementary Table 1.

### Bioinformatic analysis

Peptide spectrum matching was performed using MaxQuant (version 2.5.1.0) against the Human UniProt database (20,429 entries, December 2024; https://www.uniprot.org/proteomes/UP000005640) with TMT (Tandem Mass Tag) quantification and reporter ion distribution correction^[12]^. Search parameters included tryptic digestion with cleavage after lysine (K) and arginine (R) residues, allowing up to two missed cleavages. Output was filtered to a 1% false discovery rate at both the peptide and protein levels. Cysteine carbamidomethylation was included as a fixed modification, while methionine oxidation, asparagine and glutamine deamidation, as well as serine, threonine, and tyrosine phosphorylation were set as variable modifications.

Proteomics downstream differential expression analysis was performed in R (R Project for Statistical Computing) using the in-house package autonomics (version 1.11.100^[13]^). In brief, the MaxQuant output table “proteinGroups.txt” was imported into the R environment, and corrected reporter intensities were normalized by the reference channel intensity. Subsequently, median subtraction normalization (sample centering) was applied to all samples. Differential abundance of protein groups between conditions was evaluated by Autonomics employing a Bayesian moderated *t*-test as implemented by limma and “replicate” as a random effect, to properly account for the TMT batch^[14]^.

### ELISA

Concentrations of RAB14, SCAMP3, and FAM3C in ascitic fluid of HGSOC patients [inculde number 40 (all other information are alreadys listetd in the Supplementary Table 1, Patients_ascites)] were determined using enzyme-linked immunosorbent assay (ELISA) Kits from AFG Bioscience. FKBP4 and TGM2 in ascites were quantified by ELISA kits purchased from ELK Biotechnology, and VPS35 from Abbexa. All ELISA kits were used according to the manufacturer’s instructions. Groups were compared using the Wilcoxon rank-sum test; Kaplan-Meier survival curves for patients with high (red) *vs*. low (blue) EV abundance of RAB14, SCAMP3, and FAM3C.

### Statistics

**Figure 1:**
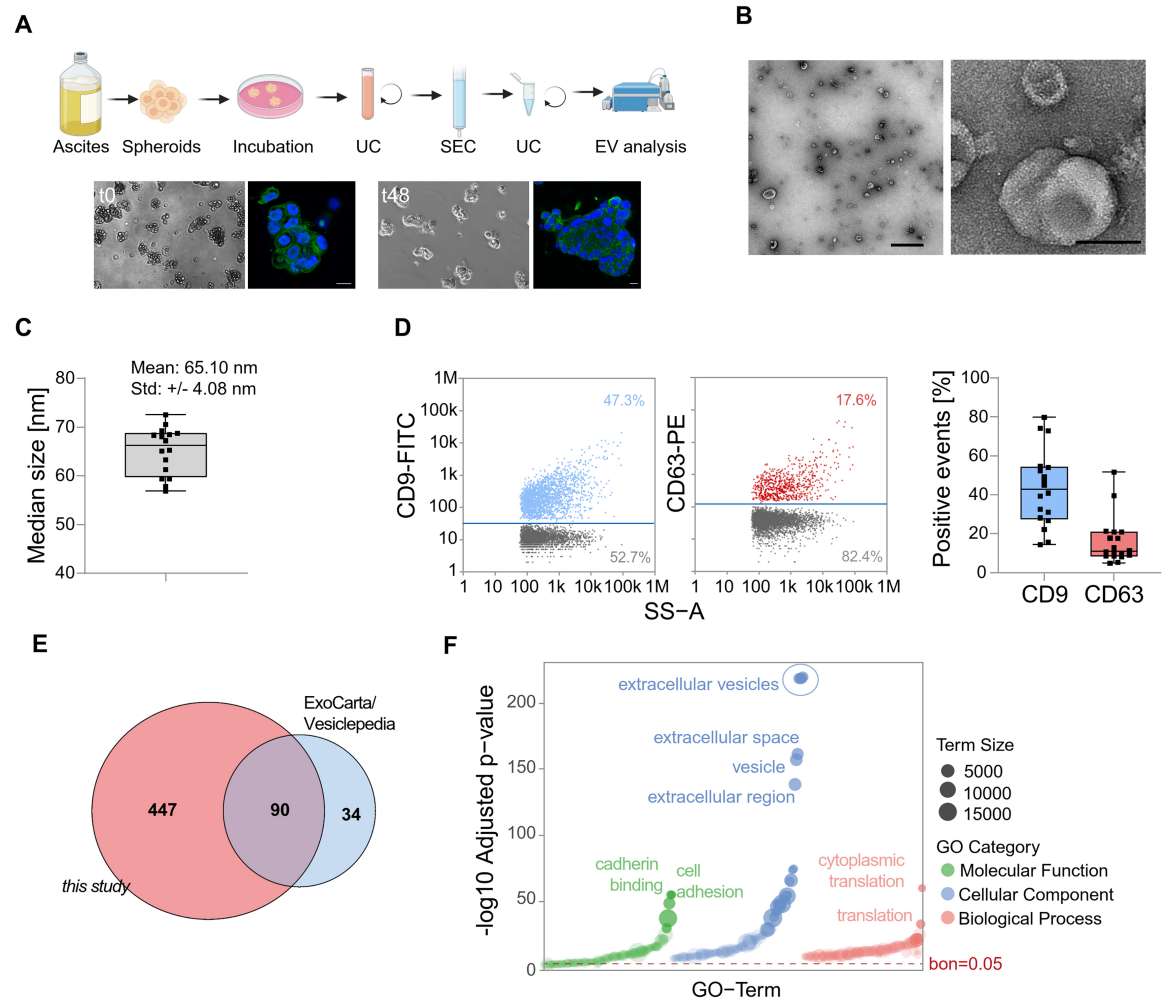
Characterization of EVs derived from HGSOC patients. (A) Spheroids were directly isolated from the ascites of HGSOC patients and subsequently cultured before EV collection. Then, EVs were isolated from the conditioned medium. Representative brightfield and fluorescence microscopy images show spheroid structures with DAPI-stained nuclei (blue) and microtubules (green). Scale bar: 100 µm; (B) Characterization of isolated EVs by TEM. Representative images are shown with scale bars of 1 µm (left) and 100 nm (right); (C) Median size of particles of EVs analyzed by nFC; (D) Single-particle phenotyping by nFC. EVs were fluorescently labeled with FITC-conjugated antibodies specific to CD9 and PE-conjugated CD63. Bivariate dot plots of indicated fluorescence *vs*. SS-A are shown. In addition, the percentage of CD9 and CD63 positive particles is depicted; (E) A Venn diagram displaying the overlap between EV proteins identified in this study and entries from ExoCarta and Vesiclepedia databases; (F) GO term enrichment analysis of proteins identified in EVs isolated from ovarian cancer spheroids. The bar plot shows the top enriched terms grouped by GO category: Cellular Component, Biological Process, and Molecular Function. Enrichment significance is indicated by -log_10_-adjusted P-values. Dashed line indicates multiple testing threshold (Bonferroni-corrected P = 0.05). EVs: Extracellular vesicles; HGSOC: high-grade serous ovarian cancer; DAPI: 4’,6-diamidino-2-phenylindole; TEM: transmission electron microscopy; nFC: nanoflow cytometry; FITC: fluorescein isothiocyanate; PE: phycoerythrin; SS-A: side scatter area; GO: gene ontology; UC: ultracentrifugation; SEC: size-exclusion chromatography.

All other statistical analyses were performed using R (version 2024.12.1) and GraphPad Prism 9. Group comparisons were conducted using unpaired two-tailed Wilcoxon rank-sum tests (unless otherwise specified). For an enrichment analysis of EV-associated danger signals, both mean peptide counts per protein across replicates [Figure 1] and log2-transformed, normalized TMT intensities [Supplementary Figure 1] were compared against all other detected proteins using two-tailed Wilcoxon rank-sum tests (without PFS stratification). Survival analyses were performed using Kaplan-Meier estimators and compared by log-rank tests. Differential protein expression was assessed by moderated t-tests with multiple testing correction (Benjamini-Hochberg method). Intensity values from proteomic analyses were log2-transformed and normalized prior to statistical evaluation. Outliers were identified and excluded based on the 1.5 × interquartile range (IQR) criterion unless stated otherwise.

GEPIA2 expression analysis of the top 3-gene signature was based on matched normal and tumor tissue data from The Cancer Genome Atlas (TCGA; https://www.cancer.gov/ccg/research/genome-sequencing/tcga) and Genotype-Tissue Expression (GTEx; https://gtexportal.org/home/) datasets. Survival analysis in GEPIA2 was performed using the 75% and 25% expression quantiles as cut-offs for high *vs*. low expression groups. For all analyses, *P* < 0.05 was considered statistically significant.

## RESULTS AND DISCUSSION

**Figure 2:**
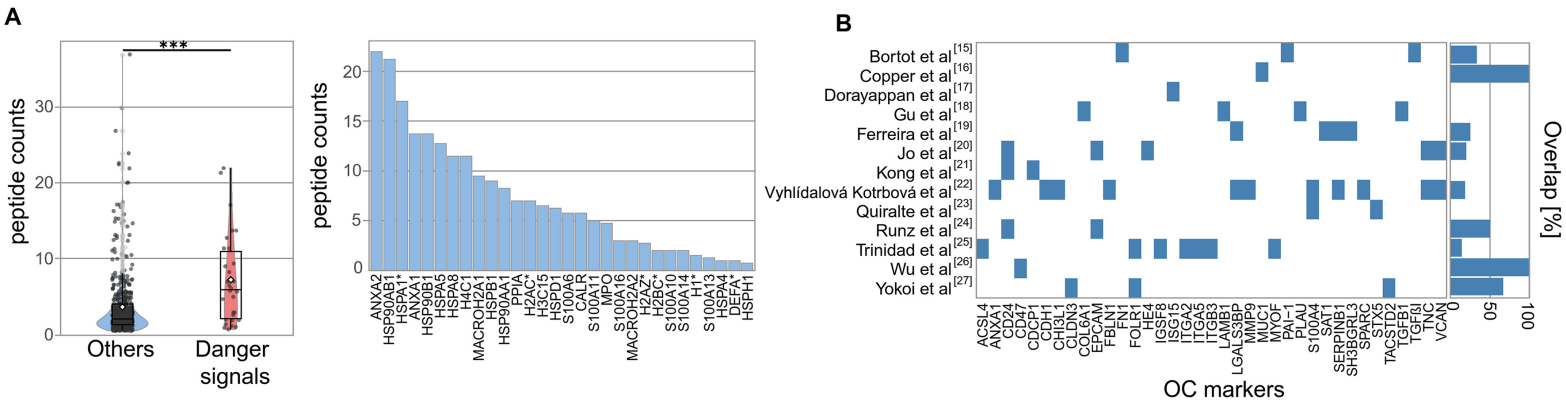
Enrichment of Danger Signals in EVs. (A) Box-and-whisker plot comparing the peptide counts of danger molecules *vs*. other proteins. Boxes = interquartile range; line = median; white dot = mean; whiskers = 1.5 × IQR; light dots = outliers. Statistical significance is indicated as ^***^P < 0.001, calculated using two-sided Wilcoxon rank-sum tests; (B) Overlap of HGSOC EV markers from published studies with the current dataset, shown as percentage per study. EVs: Extracellular vesicles; HGSOC: high-grade serous ovarian cancer; IQR: interquartile range.

The proteomic profile of EVs from primary HGSOC remains largely unexplored. Here, we characterized tumor cell-derived EVs from primary ascites spheroids (TEVs) of 23 high-grade serous ovarian cancer (HGSOC) patients [Supplementary Table 1] for in-depth proteomic profiling. Initially, the integrity and 3D architecture of the cultivated spheroids were confirmed [Figure 1A]. TEVs were isolated using a combination of differential ultracentrifugation and size-exclusion chromatography. Transmission electron microscopy (TEM) and nFC confirmed the successful isolation of small EVs from spheroids derived from HGSOC patient samples [Figure 1B-D]. Some studies of bulk ascites EVs have been published, which we compared with our dataset [summarized in Figure 2A and listed in Figure 2B].

Using mass spectrometry-based proteomic analysis, we identified 537 proteins across all replicates [Supplementary Table 1]. This list contained 90 out of 124 most frequently detected proteins in EV studies obtained from Vesiclepedia and ExoCarta [Figure 1E], indicating that EVs were efficiently isolated. In line, a gene ontology (GO) analysis revealed a highly significant enrichment of extracellular vesicle components within the cellular component domain [Figure 1F]. Moreover, the enrichment of terms associated with cell adhesion, signaling, and protein synthesis showed that spheroid-derived EVs carried tumor-relevant protein cargo.

One striking and novel feature of the TEV proteome was the high number of danger signals, known to alert the innate immune system to cellular stress^[28]^, specifically heat shock proteins, calcium-binding S100 family members, and extracellular histones (Wilcoxon test, *P* = 5.8 × 10^-5^; peptide count comparison, Figure 2A). Interestingly, histones are emerging EV-associated surface proteins, contributing to cellular stress responses and vesicle function or uptake^[29]^. It is well established that sustained exposure to danger signals triggers chronic inflammation and contributes to the establishment of a tumor-promoting microenvironment. Elevated levels have been associated with poor prognosis in ovarian cancer, and targeting alarmin receptors (e.g., TLR4, RAGE) is a potential therapeutic strategy^[30]^.

**Figure 3:**
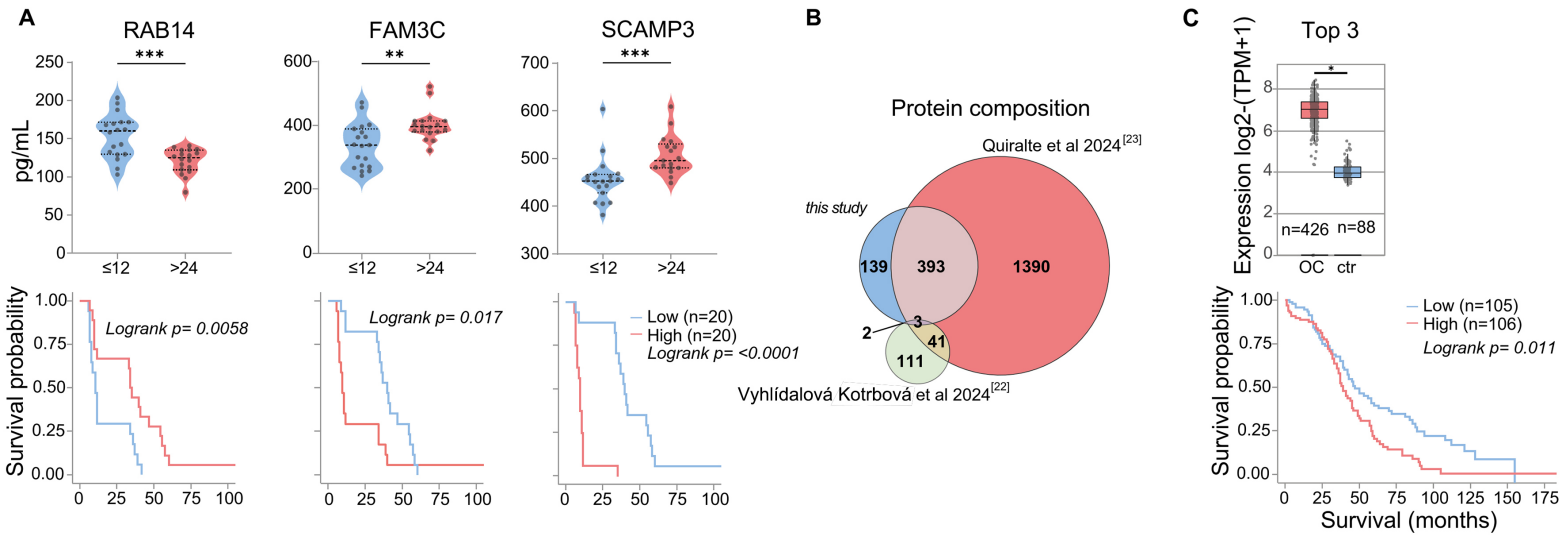
Survival-Linked EV Signatures. (A) ELISA quantification and Kaplan-Meier analysis of RAB14, FAM3C, and SCAMP3 expression. Statistical significance is indicated as ^**^P < 0.01; and ^***^P < 0.001, calculated using two-sided Wilcoxon rank-sum tests; (B) Comparison of EV protein detection across three independent ovarian cancer EV datasets; (C) GEPIA2 mRNA expression TCGA analysis of selected markers (CORO1B, LAMP2, MSLN) in tumor samples *vs*. matched healthy samples and GTEx data (boxes = interquartile range; line = median). Statistical significance: ^*^P < 0.05, calculated by one-way ANOVA on log2(TPM + 1)-transformed data. The corresponding GEPIA2 Kaplan-Meier plot with a 75% high-expression cut-off is shown (log-rank test; details see Supplementary Figure 2). EV: Extracellular vesicle; ELISA: enzyme-linked immunosorbent assay; GEPIA2: Gene Expression Profiling Interactive Analysis 2; TCGA: The Cancer Genome Atlas; GTEx: Genotype-Tissue Expression; IQR: interquartile range; ANOVA: analysis of variance; TPM: transcripts per million.

The prevalence of danger molecules among ascites TEV-associated proteins has not been observed so far for total ascites EVs, although some of these candidate biomarkers were previously identified in TEVs and also in ascites EV datasets [Figure 2B]. To summarize, our approach resulted in the detection of several already known clinically relevant proteins, which are overexpressed in HGSOC and associated with shorter survival (e.g., PODXL, MUC1, MIF, CXCL10, HSP70), and we identified a previously unreported enrichment of EV-associated danger molecules. The systemic and local impact of TEVs on tumor progression and outcome remains to be investigated. Given the immunosuppressive nature of malignant ascites, we hypothesize that spheroid-derived EVs may contribute to the local immune regulation, potentially influencing MDSCs (myeloid-derived suppressor cells) and other suppressor populations^[31]^. A statistically robust quantification of TEV-associated proteins in patients with longer *vs*. shorter survival was not possible due to technical variability and limited cohort size (*n* = 23). However, a subset of proteins showed a trend for differential abundance [Supplementary Figure 1A]. Six candidates from this group (RAB14: less in patients with shorter survival; SCAMP3, FAM3C, FKBP4, TGM2, and VPS35: less in patients with longer survival) were selected for validation based on prior evidence linking them to cancer progression or EV biogenesis. ELISA results from patient ascites confirmed significantly different abundance levels of three factors in patients with PFS shorter than 12 months compared to those with PFS longer than 24 months [Figure 3A and Supplementary Figure 1B]. This indicates that TEVs harbor previously unrecognized clinically relevant proteins.

To specifically identify novel tumor-cell released proteins, we compared our dataset with two recently published studies on patient ascites or peritoneal fluid EVs^[22,23]^, one of them additionally filtered for patient-specific proteins^[22]^. In total, 537 proteins detected in the TEV samples were also detected in these studies, meaning that our study allocates 139 novel proteins to tumor cell-derived EVs [Figure 3B]. This reflects the observation that only a fraction of ascites EVs is released by tumor cells^[22]^, and bulk analyses can only partly unravel the molecular composition of TEVs. We hypothesize that these TEV-associated specific proteins may provide an additional, so far underexplored source for TME and biomarker research.

We used the GEPIA2 software to address the potential clinical relevance of novel TEV-associated factors and filtered the spheroid-specific 139 EV protein list for survival-associated gene expression (GEPIA2). TCGA and GTEx RNA-Seq data revealed that a signature of the top three genes (CORO1B, LAMP2, MSLN) was overexpressed in ovarian tumor tissue and correlated significantly with shorter overall survival [Figure 3C and Supplementary Figure 2]. Among these, MSLN is known as an ovarian cancer tumor-associated antigen^[32]^. The clinical significance of MSLN in association with EVs and the diagnostic potential of the EV-HGSOC signature will be assessed in an independent cohort for further validation.

In conclusion, our study defines novel clinically relevant ovarian cancer-associated proteins, which are released from tumor cells via EVs. The spheroid EV proteome dataset may also provide a foundation for mechanistic studies to better understand molecular pathways contributing to tumor pathogenesis in the TME.

## DECLARATIONS

### Availability of data and materials

All data generated or analyzed during this study are included in this published article and its Supplementary Materials.

### AI and AI-assisted tools statement

Artificial intelligence tools (ChatGPT, OpenAI) were used for spell checking and minor assistance with R code formatting.

### Copyright

© The Author(s) 2025.
