# Supplementary material for: Extracellular vesicles from ovarian cancer tumor spheroids harbor disease-related and survival-associated proteins
Source: Extracell Vesicles Circ Nucl Acids. 2025 Nov 5;6(4):677–86. doi: 10.20517/evcna.2025.70 (PMC12809391; doi:10.20517/evcna.2025.70)
Supplement: Supplementary file 1 [file evcna-6-4-677-SupplementaryMaterials.zip › evcna6070-SupplementaryMaterials/evcna6070-SupplementaryMaterials.pdf]

Supplementary Materials

Extracellular vesicles from ovarian cancer tumor spheroids harbor disease-related and survival-associated proteins

Christian Preußer[1,2], Max Gläser[1], Johannes Graumann[3], Witold Szymański[3], Daniel Bachurski[4], María Gómez-Serrano[1], Ralf Jacob[5], Silke Reinartz[1], Elke Pogge von Strandmann[1,2]

[1]Institute for Tumor Immunology, Philipps University Marburg, Marburg 35043, Germany.
[2]EV-iTEC Core Facility, Philipps University Marburg, Marburg 35043, Germany.
[3]Institute of Translational Proteomics and Core Facility Translational Proteomics, Philipps University Marburg, Marburg 35043, Germany.
[4]Cluster of Excellence on Cellular Stress Responses in Aging-Associated Diseases (CECAD), University of Cologne, Cologne 50931, Germany.
[5]Department of Cell Biology and Cell Pathology, Philipps University Marburg, Marburg 35043, Germany.

Correspondence to: Prof. Elke Pogge von Strandmann, Institute for Tumor Immunology, Philipps University Marburg, Marburg 35043, Germany. E-mail: poggevon@staff.uni-marburg.de

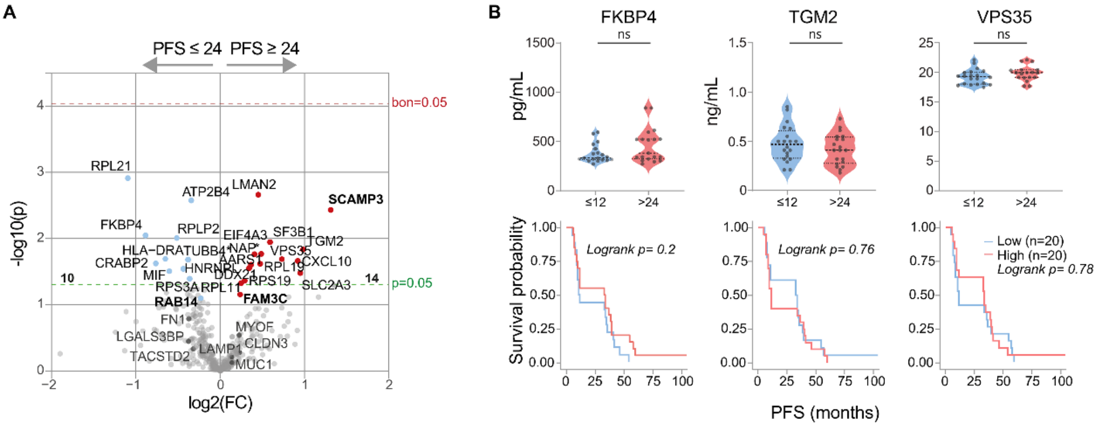

Supplementary Figure 1. (A) Exploratory volcano plot showing differential EV protein abundance in patients with short (≤ 24 months) versus long (≥ 24 months) progression-free survival (PFS). The log2 fold changes and *P*-values shown are based on TMT reporter ion intensities, based on median subtraction normalization (sample centering) to compensate for sample variability; (B) Association of selected EV proteins with PFS and protein concentration**.** Protein concentrations of VPS35, FKBP4, and TGM2 were measured by ELISA in ascites-derived EVs from patients with PFS ≤ 12 months *vs*. > 24 months. Groups were compared using the Wilcoxon rank-sum test; ns = not significant. Kaplan-Meier survival curves for patients with high (red) *vs*. low (blue) EV abundance of VPS35, FKBP4, and TGM2. Patients were compared by median expression level; *P*-values were calculated using the log-rank test.

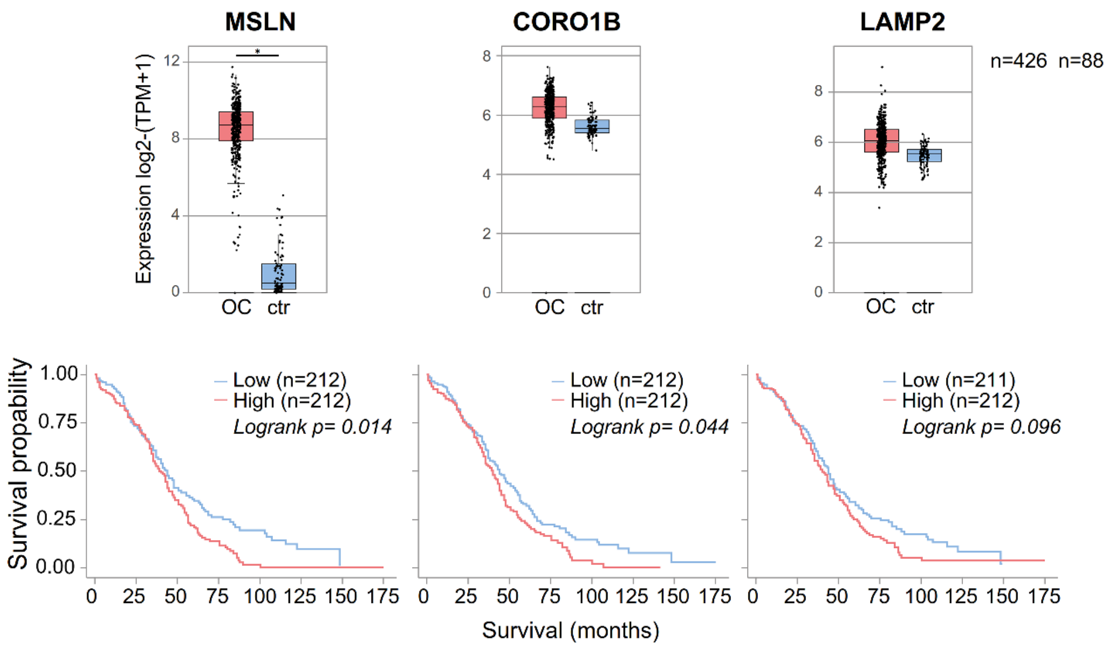

Supplementary Figure 2. Expression of MSLN, CORO1B, and LAMP2 in OC patients versus healthy controls (TCGA/GTEx) and corresponding Kaplan-Meier survival plots (75% cut-off, GEPIA2). Statistical significance: $P < 0.05$ (*), calculated by one-way ANOVA on log2(TPM+1)-transformed data.
